# Crystal structure of 2-(2,6-diiso­propyl­phen­yl)-*N*,*N*-diethyl-3,3-dimethyl-2-aza­spiro­[4.5]decan-1-amine: a di­ethyl­amine adduct of a cyclic(alk­yl)(amino)­carbene (CAAC)

**DOI:** 10.1107/S2056989021007854

**Published:** 2021-08-06

**Authors:** Roxanne A. Naumann, Joseph W. Ziller, Allegra L. Liberman-Martin

**Affiliations:** a Chemistry and Biochemistry Program, Schmid College of Science and Technology, Chapman University, 1 University Drive, Orange, CA 92866, USA; b Department of Chemistry, University of California, Irvine, Natural Sciences II, Irvine, CA 92697, USA

**Keywords:** crystal structure, cyclic(alk­yl)(amino)­carbene, carbene reactivity, N—H bond activation

## Abstract

The structure of the title compound, C_27_H_46_N_2_, at 93 K has monoclinic (*P*2_1_/*n*) symmetry. The title compound was prepared by treatment of 2-(2,6-diiso­propyl­phenyl)-3,3-dimethyl-2-aza­spiro­[4.5]dec-1-en-2-ium hydrogen dichloride with two equivalents of lithium di­ethyl­amide. Characterization of the title compound by single-crystal X-ray diffraction and ^1^H and ^13^C NMR spectroscopy is presented. Formation of the di­ethyl­amine adduct of the cyclic(alk­yl)(amino)­carbene (CAAC) was unexpected, as deprotonation using lithium diiso­propyl­amide results in free CAAC formation.

## Abstract 2

A di­ethyl­amine adduct of a cyclic(alk­yl)(amino)­carbene was synthesized and characterized by single-crystal X-ray diffraction and NMR spectroscopy.

## Chemical context   

Cyclic (alk­yl)(amino)­carbenes (CAACs) are a class of singlet carbenes featuring a carbene center flanked by one amino substituent and an alkyl substituent. Compared to *N*-heterocyclic carbenes (NHCs), CAACs are simultaneously stronger σ-donors and stronger π-acceptors (Melaimi *et al.*, 2017 ▸). These properties, along with the distinctive CAAC steric environment imparted by the quaternary carbon adjacent to the carbene center, have enabled the application of CAACs as ligands to stabilize unusual transition-metal (Roy *et al.*, 2016 ▸) and main-group compounds (Soleilhavoup & Bertrand, 2015 ▸).

As a result of their strong electrophilicity, CAACs are capable of activating strong bonds, including H—H, N—H, P—H, Si—H, and B—H bonds (Frey *et al.*, 2007 ▸, 2010 ▸). Analogous oxidative addition reactivity has also been observed for other classes of electrophilic carbenes, including *N*,*N*′-di­amido­carbenes (DACs) (Hudnall *et al.*, 2010 ▸; Moerdyk *et al.*, 2013 ▸; Chase *et al.*, 2014 ▸; Lastovickova & Bielawski, 2016 ▸). Despite this rich reactivity, these carbene oxidative addition reactions are typically irreversible. However, a recent report demonstrated that a CAAC with a sterically demanding menthol-derived quaternary carbon substituent undergoes N—H and P—H reductive eliminations (Tolentino *et al.*, 2019 ▸). This established the reversibility of oxidative addition and reductive elimination reaction at carbon centers, and suggests that CAACs and other electrophilic carbenes may be able to perform catalytic coupling reactions.

**Figure 1:**
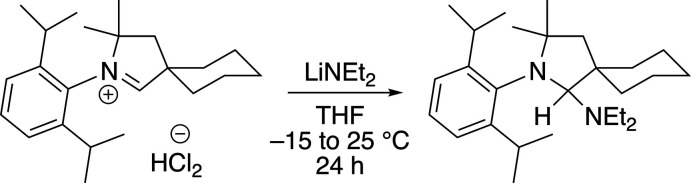
Synthesis of the title compound.

In the current work, we report the structure of the title compound 2-(2,6-diiso­propyl­phenyl)-*N*,*N*-diethyl-3,3-di­meth­yl-2-aza­spiro­[4.5]decan-1-amine, which was prepared by treatment of 2-(2,6-diiso­propylphenyl)-3,3-dimethyl-2-aza­spiro­[4.5]dec-1-en-2-ium hydrogen dichloride with two equivalents of lithium di­ethyl­amide (Fig. 1 ▸). Previous syntheses to furnish the corresponding free CAAC carbene have employed the bulkier bases lithium diiso­propyl­amide or potassium bis­(tri­methyl­sil­yl)amide (Lavallo *et al.*, 2005 ▸; Tolentino *et al.*, 2019 ▸), indicating that the size of the amine significantly impacts the propensity toward amine addition to the CAAC.
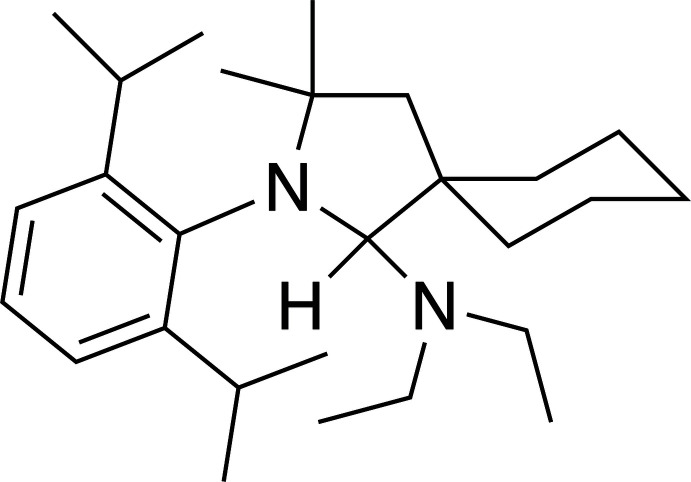


## Structural commentary   

**Figure 2:**
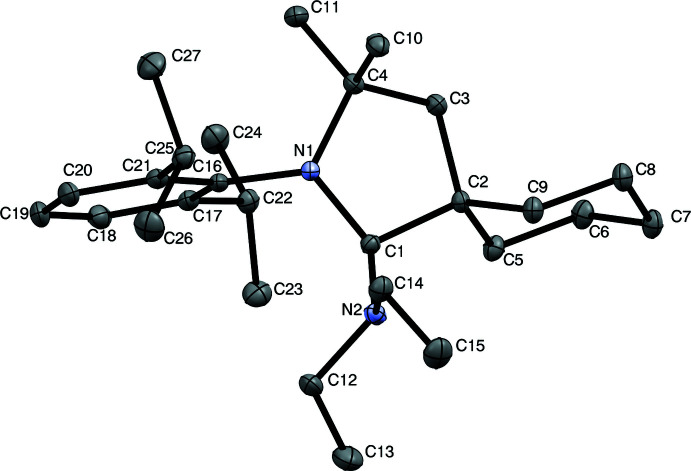
Mol­ecular structure of the title compound. Displacement ellipsoids are drawn at the 50% probability level. H atoms are omitted for clarity.

The mol­ecular structure of the title compound is presented in Fig. 2 ▸, and selected geometric parameters are summarized in Table 1 ▸. As expected, X-ray diffraction analysis confirmed the pyramidalization of the former carbene carbon center (the sum of the N1—C1—C2, N1—C1—N2, and C2—C1—N2 angles is 337.79°), consistent with *sp* ^3^ hybridization. The C–NEt_2_ (C1—N2) bond length [1.4675 (14) Å] is a typical distance for a carbon–nitro­gen single bond (Allen *et al.*, 1987 ▸) and is similar to bond distances observed for previously reported CAAC N–H insertion products (see Database Survey section below).

The cyclo­hexyl ring (C2, C5–C9) of the title compound adopts a chair conformation. The cyclic nitro­gen atom, N1, is distorted from planarity (sum of C1—N1—C4, C1—N1—C16, and C4—N1—C16 angles = 351.21°). This differs from the analogous CAAC structure in its free carbene form, in which there is π-donation from nitro­gen to stabilize the carbene center (sum of bond angles around nitro­gen = 356.57°; Frey *et al.*, 2007 ▸). Bond angles for the di­ethyl­amino nitro­gen atom, N2, are consistent with *sp* ^3^ hybridization (sum of C1—N2—C12, C1—N2—C14, and C12—N2—C14 angles = 343.51°).

## Supra­molecular features   

**Figure 3:**
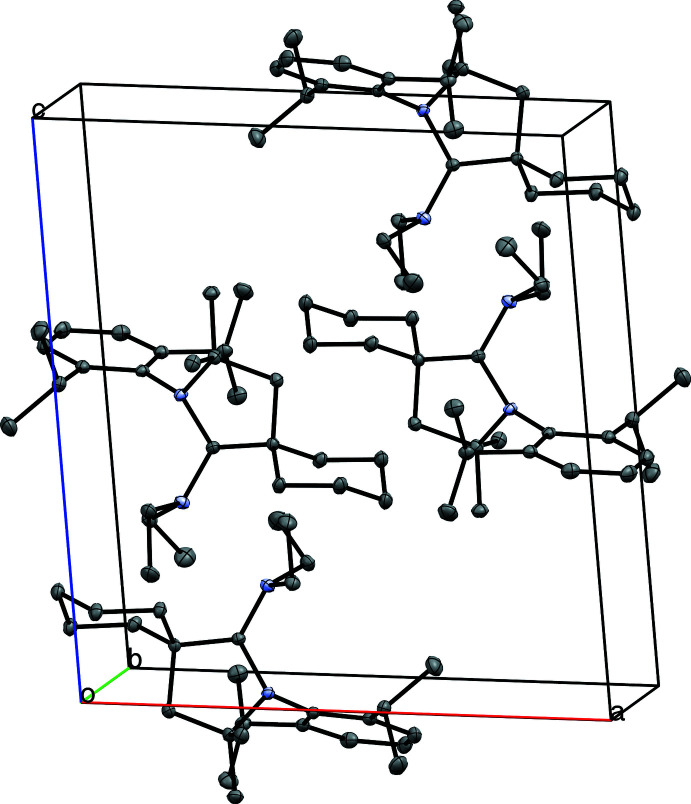
View of the four mol­ecules of the title compound in the unit cell. Displacement ellipsoids are drawn at the 50% probability level. H atoms are omitted for clarity.

**Figure 4:**
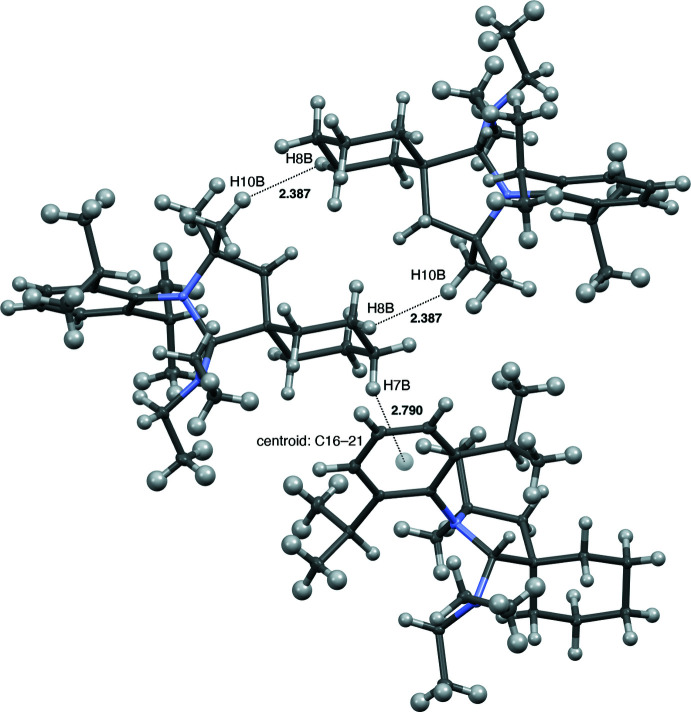
View of short inter­molecular distances between neighboring mol­ecules of the title compound. Displacement ellipsoids are drawn at the 50% probability level.

Four mol­ecules of the title compound are present in the unit cell, as shown in Fig. 3 ▸. The methyl­ene group at the 4-position of the cyclo­hexyl ring is oriented towards the aryl ring of the 2,6-diiso­propyl­phenyl group of the neighboring mol­ecule, with a distance of 2.790 Å between the aryl ring centroid (C16–C21) and the nearest methyl­ene hydrogen atom (H7*B*). The mol­ecules are oriented such that the 3-position of the cyclo­hexyl ring of one mol­ecule is adjacent to a methyl group at the gem-dimethyl position of a neighboring mol­ecule, with a distance of 2.387 Å between nearest hydrogen atoms (H8*B* · · · H10*B*) (Fig. 4 ▸).

## Database survey   

A survey of the Cambridge Structural Database (2020 Version, *ConQuest* 2.0.5; Groom *et al.*, 2016 ▸) was performed to identify structures of related free CAAC and CAAC amine addition compounds. The crystal structure for the corresponding CAAC free carbene has been reported (CSD refcode GIDWAS; Frey *et al.*, 2007 ▸). Crystal structures have also been reported for CAAC N—H bond-activation products involving ammonia (CSD refcode GIDWEW; Frey *et al.*, 2007 ▸), di­phenyl­amine (CSD refcodes GOMGEX and GOMGUN; Tolentino *et al.*, 2019 ▸), imidazole (CSD refcode RARHOK; Paul & Radius, 2017 ▸), benzimidazole (CSD refcode RARHUQ; Paul & Radius, 2017 ▸), 2-phenyl­benzimidazole (CSD refcode HOKTAF; Kieser *et al.*, 2019 ▸), and carbazole (CSD refcode HOKOS; Kieser *et al.*, 2019 ▸).

## Synthesis and crystallization   

The synthesis of 2-(2,6-diiso­propylphenyl)-*N*,*N*-diethyl-3,3-dimethyl-2-aza­spiro­[4.5]decan-1-amine is summarized in Fig. 1 ▸. All solvents were dried by passage through solvent purification columns (JC Meyer) and stored over activated 3 Å mol­ecular sieves. Lithium di­ethyl­amide (Sigma–Aldrich) and 2-(2,6-diiso­propylphenyl)-3,3-dimethyl-2-aza­spiro­[4.5]dec-1-en-2-ium hydrogen dichloride (TCI America) were used as received. Celite (Aldrich) was dried under vacuum at 473 K for 48 h before use. The ^1^H and ^13^C NMR spectra were recorded on a Bruker AVANCE III 400 MHz NMR spectrometer at 298 K. The ^1^H NMR spectrum was calibrated inter­nally to resonances for the residual proteo solvent relative to tetra­methyl­silane. The ^13^C NMR spectrum was calibrated to the solvent resonance relative to tetra­methyl­silane. Spectra were analyzed using *MestReNova* Ver. 14.2.0 software.

In a nitro­gen-atmosphere glovebox, a cold (258 K) solution of lithium di­ethyl­amide (99 mg, 1.3 mmol) in 8 mL of THF was added dropwise to a stirred cold (258 K) suspension of 2-(2,6-diiso­propylphenyl)-3,3-dimethyl-2-aza­spiro­[4.5]dec-1-en-2-ium hydrogen dichloride (250 mg, 0.63 mmol) in 5 mL of THF. The orange solution was slowly warmed to room temperature. After 24 h, volatiles were removed *in vacuo* to afford a pale orange solid. After extraction with pentane (2 × 20 mL) and filtration through Celite on a fritted funnel, evaporation of volatiles *in vacuo* afforded a pale orange solid. Single crystals suitable for X-ray analysis were grown by slow evaporation of a pentane solution of the crude product at 258 K, which led to the formation of colorless block-like crystals of the title compound (115 mg, 46% yield).

^1^H NMR (400 MHz, benzene-*d*_6_, 198 K): δ = 7.04–7.13 (*m*, 3H, H_Ar_), 4.46 (*s*, 1H, CAAC CH), 3.98 (*sept*, 1H, C*H*(CH_3_)_2_, *J* = 6.9 Hz), 3.17 (*sept*, 1H, C*H*(CH_3_)_2_, *J* = 6.9 Hz), 2.54 (*br s*, 4H), 2.15 (*d*, 1H, *J* = 13.7 Hz, diastereotopic C*H*_2_), 1.97 (*d*, 1H, *J* = 12.7 Hz, diastereotopic C*H*­_2_), 1.49–1.87 (*m*, 10H), 1.47 (*s*, 2H), 1.30–1.39 (*m*, 6H, CH(C*H*_3_)_2_), 1.21 (*m*, 6H, CH(C*H*_3_)_2_), 0.80–1.06 (*br m*, 6H), 0.95 (*s*, 3H), 0.86 (*t*, 2H, *J* = 7.1 Hz). ^13^C NMR (101 MHz, benzene-*d*_6_, 298 K): δ = 151.02 (aryl C_quat_), 148.36 (aryl C_quat_), 144.33 (aryl C_quat_), 126.58(aryl CH), 125.81 (aryl CH), 124.64 (aryl CH), 97.44 [*C*H(NEt_2_)], 61.30 (C_quat_), 51.57 (CH_2_), 45.73 (C_quat_), 41.26 (CH_2_), 32.99 (CH_3_), 32.77 (CH_2_), 29.15 (CH_3_), 28.96 [*C*H(CH_3_)_2_], 27.45 [*C*H(CH_3_)_2_], 26.98 (CH_3_), 26.61 (CH_2_), 25.58 (CH_3_), 25.05 (CH_3_), 25.03 (CH_2_), 24.86 (CH_3_), 23.71 (CH_2_).

## Refinement   

Crystal data, data collection and structure refinement details are summarized in Table 2 ▸. Hydrogen atoms were included using a riding model, with C—H = 0.95–1.00 Å and *U*_iso_(H) = 1.2*U*_eq_(C) or 1.5*U*_eq_(C-meth­yl).

## Supplementary Material

Crystal structure: contains datablock(s) I. DOI: 10.1107/S2056989021007854/zl5021sup1.cif

Structure factors: contains datablock(s) I. DOI: 10.1107/S2056989021007854/zl5021Isup2.hkl

Supporting information file. DOI: 10.1107/S2056989021007854/zl5021Isup3.cml

CCDC reference: 2100575

Additional supporting information:  crystallographic information; 3D view; checkCIF report

## Figures and Tables

**Table 1:** Selected geometric parameters (Å, °)

Bond distances		
C_carbene_—NEt_2_	C1—N2	1.4675 (14)
C_carbene_—N_cyclic_	C1—N1	1.4794 (15)
C_carbene_—C_spiro_	C1—C2	1.5741 (16)
N_cyclic_—CMe_2_	N1—C4	1.4928 (15)
C_spiro_—CH_2_	C2—C3	1.5449 (16)
CMe_2_—CH_2_	C4—C3	1.5402 (16)
Bond angles		
N_cyclic_—C_carbene_—C_spiro_	N1—C1—C2	106.30 (9)
N_cyclic_—C_carbene_—NEt_2_	N1—C1—N2	116.25 (9)
C_spiro_—C_carbene_—NEt_2_	C2—C1—N2	115.24 (9)
C_carbene_—N_cyclic_—C_Dipp_	C1—N1—C16	117.52 (9)

**Table 2:** Experimental details

Crystal data	
Chemical formula	C_27_H_46_N_2_
*M*_r_	398.66
Crystal system, space group	Monoclinic, *P*2_1_/*n*
Temperature (K)	93
*a*, *b*, *c* (Å)	12.3319 (11), 14.4082 (13), 13.6155 (12)
β (°)	96.4589 (16)
*V* (Å^3^)	2403.9 (4)
*Z*	4
Radiation type	Mo *K*α
μ (mm^−1^)	0.06
Crystal size (mm)	0.25 × 0.25 × 0.19

Data collection	
Diffractometer	Bruker SMART APEXII CCD
Absorption correction	Multi-scan (*SADABS*; Bruker, 2014 ▸)
*T*_min_, *T*_max_	0.823, 0.862
No. of measured, independent and observed [*I* > 2σ(*I*)] reflections	57200, 6744, 5087
*R*_int_	0.069
(sin θ/λ)_max_ (Å^−1^)	0.694

Refinement	
*R*[*F*^2^ > 2σ(*F* ^2^)], *wR*(*F* ^2^), *S*	0.048, 0.121, 1.02
No. of reflections	6744
No. of parameters	270
H-atom treatment	H-atom parameters constrained
Δρ_max_, Δρ_min_ (e Å^−3^)	0.38, −0.25

Computer programs: *APEX2* and *SAINT* (Bruker, 2014 ▸), *SHELXT2014/4* (Sheldrick, 2015*a* ▸), *SHELXL2014/7* (Sheldrick, 2015*b* ▸), and *SHELXTL* (Sheldrick, 2008 ▸).

## supplementary crystallographic information

### Crystal data

**Table:**

C_27_H_46_N_2_	*F*(000) = 888
*M**_r_* = 398.66	*D*_x_ = 1.102 Mg m^−^^3^
Monoclinic, *P*2_1_/*n*	Mo *K*α radiation, λ = 0.71073 Å
*a* = 12.3319 (11) Å	Cell parameters from 9991 reflections
*b* = 14.4082 (13) Å	θ = 2.2–30.5°
*c* = 13.6155 (12) Å	µ = 0.06 mm^−^^1^
β = 96.4589 (16)°	*T* = 93 K
*V* = 2403.9 (4) Å^3^	Irregular, colorless
*Z* = 4	0.25 × 0.25 × 0.19 mm

### Data collection

**Table:**

Bruker SMART APEXII CCD diffractometer	5087 reflections with *I* > 2σ(*I*)
Radiation source: fine-focus sealed tube	*R*_int_ = 0.069
φ and ω scans	θ_max_ = 29.6°, θ_min_ = 2.1°
Absorption correction: multi-scan (SADABS; Bruker, 2014)	*h* = −17→17
*T*_min_ = 0.823, *T*_max_ = 0.862	*k* = −20→20
57200 measured reflections	*l* = −18→18
6744 independent reflections	

### Refinement

**Table:**

Refinement on *F*^2^	Primary atom site location: structure-invariant direct methods
Least-squares matrix: full	Secondary atom site location: difference Fourier map
*R*[*F*^2^ > 2σ(*F*^2^)] = 0.048	Hydrogen site location: inferred from neighbouring sites
*wR*(*F*^2^) = 0.121	H-atom parameters constrained
*S* = 1.02	*w* = 1/[σ^2^(*F*_o_^2^) + (0.0481*P*)^2^ + 1.1815*P*] where *P* = (*F*_o_^2^ + 2*F*_c_^2^)/3
6744 reflections	(Δ/σ)_max_ < 0.001
270 parameters	Δρ_max_ = 0.38 e Å^−^^3^
0 restraints	Δρ_min_ = −0.25 e Å^−^^3^

### Special details

**Table:**

Geometry. All esds (except the esd in the dihedral angle between two l.s. planes) are estimated using the full covariance matrix. The cell esds are taken into account individually in the estimation of esds in distances, angles and torsion angles; correlations between esds in cell parameters are only used when they are defined by crystal symmetry. An approximate (isotropic) treatment of cell esds is used for estimating esds involving l.s. planes.
Refinement. A colorless crystal of approximate dimensions 0.191 x 0.248 x 0.250 mm was mounted in a cryoloop and transferred to a Bruker SMART APEX II diffractometer. The APEX2 program package was used to determine the unit-cell parameters and for data collection (25 sec/frame scan time). The raw frame data was processed using SAINT and SADABS to yield the reflection data file. Subsequent calculations were carried out using the SHELXTL program package. The diffraction symmetry was 2/m and the systematic absences were consistent with the monoclinic space group P21/n that was later determined to be correct.The structure was solved by direct methods and refined on F2 by full-matrix least-squares techniques. The analytical scattering factors for neutral atoms were used throughout the analysis. Hydrogen atoms were included using a riding model.Least-squares analysis yielded wR2 = 0.1207 and Goof = 1.018 for 270 variables refined against 6744 data (0.72 ), R1 = 0.0482 for those 5087 data with I > 2.0sigma(I).

### Fractional atomic coordinates and isotropic or equivalent isotropic displacement parameters (Å^2^)

**Table:**

	*x*	*y*	*z*	*U*_iso_*/*U*_eq_	
N1	0.21509 (8)	0.23294 (7)	0.51919 (7)	0.0098 (2)	
N2	0.19958 (8)	0.23854 (7)	0.33444 (7)	0.0116 (2)	
C1	0.26502 (9)	0.25804 (8)	0.42898 (8)	0.0098 (2)	
H1A	0.2755	0.3269	0.4316	0.012*	
C2	0.38287 (9)	0.21455 (8)	0.44118 (8)	0.0100 (2)	
C3	0.40228 (9)	0.19144 (8)	0.55256 (8)	0.0116 (2)	
H3A	0.4473	0.1347	0.5633	0.014*	
H3B	0.4410	0.2432	0.5892	0.014*	
C4	0.28990 (9)	0.17623 (8)	0.58916 (9)	0.0112 (2)	
C5	0.46257 (10)	0.28977 (8)	0.41182 (9)	0.0127 (2)	
H5A	0.4390	0.3100	0.3433	0.015*	
H5B	0.4585	0.3442	0.4556	0.015*	
C6	0.58148 (10)	0.25689 (9)	0.41836 (9)	0.0149 (2)	
H6A	0.6277	0.3075	0.3970	0.018*	
H6B	0.6078	0.2413	0.4878	0.018*	
C7	0.59148 (10)	0.17188 (9)	0.35313 (9)	0.0149 (2)	
H7A	0.6677	0.1492	0.3614	0.018*	
H7B	0.5725	0.1890	0.2829	0.018*	
C8	0.51526 (10)	0.09514 (9)	0.38112 (9)	0.0140 (2)	
H8A	0.5397	0.0735	0.4490	0.017*	
H8B	0.5193	0.0419	0.3357	0.017*	
C9	0.39694 (9)	0.12857 (8)	0.37605 (9)	0.0122 (2)	
H9A	0.3512	0.0775	0.3973	0.015*	
H9B	0.3703	0.1438	0.3066	0.015*	
C10	0.25722 (10)	0.07300 (8)	0.58361 (9)	0.0144 (2)	
H10A	0.1848	0.0655	0.6058	0.022*	
H10B	0.3107	0.0366	0.6262	0.022*	
H10C	0.2554	0.0512	0.5152	0.022*	
C11	0.29103 (10)	0.20714 (9)	0.69696 (9)	0.0141 (2)	
H11A	0.3137	0.2723	0.7032	0.021*	
H11B	0.3424	0.1685	0.7392	0.021*	
H11C	0.2177	0.2004	0.7174	0.021*	
C12	0.12797 (10)	0.31610 (9)	0.29816 (9)	0.0152 (3)	
H12A	0.1066	0.3513	0.3554	0.018*	
H12B	0.0607	0.2908	0.2612	0.018*	
C13	0.18274 (12)	0.38151 (10)	0.23150 (11)	0.0228 (3)	
H13A	0.1331	0.4328	0.2110	0.034*	
H13B	0.2006	0.3477	0.1730	0.034*	
H13C	0.2498	0.4062	0.2675	0.034*	
C14	0.14360 (10)	0.14878 (9)	0.32281 (9)	0.0142 (2)	
H14A	0.0682	0.1555	0.3404	0.017*	
H14B	0.1821	0.1031	0.3686	0.017*	
C15	0.13992 (11)	0.11302 (10)	0.21688 (9)	0.0196 (3)	
H15A	0.1024	0.0530	0.2114	0.029*	
H15B	0.2145	0.1056	0.1997	0.029*	
H15C	0.1005	0.1575	0.1716	0.029*	
C16	0.13925 (9)	0.29785 (8)	0.55420 (8)	0.0101 (2)	
C17	0.16954 (10)	0.39033 (8)	0.58137 (8)	0.0118 (2)	
C18	0.08998 (10)	0.45134 (9)	0.60845 (9)	0.0150 (2)	
H18A	0.1097	0.5136	0.6250	0.018*	
C19	−0.01697 (10)	0.42321 (9)	0.61176 (9)	0.0167 (3)	
H19A	−0.0700	0.4659	0.6298	0.020*	
C20	−0.04562 (10)	0.33259 (9)	0.58858 (9)	0.0153 (3)	
H20A	−0.1185	0.3129	0.5927	0.018*	
C21	0.03019 (10)	0.26912 (8)	0.55924 (8)	0.0117 (2)	
C22	0.28572 (10)	0.42758 (8)	0.58449 (9)	0.0124 (2)	
H22A	0.3346	0.3746	0.5717	0.015*	
C23	0.29540 (11)	0.50154 (9)	0.50440 (9)	0.0173 (3)	
H23A	0.2704	0.4754	0.4393	0.026*	
H23B	0.3717	0.5210	0.5060	0.026*	
H23C	0.2503	0.5553	0.5169	0.026*	
C24	0.32675 (11)	0.46913 (10)	0.68579 (10)	0.0192 (3)	
H24A	0.3178	0.4235	0.7377	0.029*	
H24B	0.2845	0.5250	0.6970	0.029*	
H24C	0.4041	0.4854	0.6873	0.029*	
C25	−0.00794 (10)	0.17054 (9)	0.53442 (9)	0.0134 (2)	
H25A	0.0520	0.1375	0.5050	0.016*	
C26	−0.10981 (11)	0.16883 (10)	0.45862 (10)	0.0213 (3)	
H26A	−0.0967	0.2062	0.4010	0.032*	
H26B	−0.1720	0.1944	0.4886	0.032*	
H26C	−0.1259	0.1047	0.4378	0.032*	
C27	−0.03084 (11)	0.11760 (10)	0.62790 (10)	0.0207 (3)	
H27A	0.0339	0.1199	0.6767	0.031*	
H27B	−0.0484	0.0528	0.6109	0.031*	
H27C	−0.0926	0.1464	0.6557	0.031*	

### Atomic displacement parameters (Å^2^)

**Table:**

	*U*^11^	*U*^22^	*U*^33^	*U*^12^	*U*^13^	*U*^23^
N1	0.0099 (4)	0.0105 (5)	0.0093 (4)	0.0021 (4)	0.0023 (3)	0.0011 (4)
N2	0.0117 (5)	0.0120 (5)	0.0107 (4)	0.0014 (4)	−0.0009 (4)	−0.0001 (4)
C1	0.0103 (5)	0.0100 (5)	0.0090 (5)	0.0001 (4)	0.0015 (4)	−0.0003 (4)
C2	0.0083 (5)	0.0106 (5)	0.0111 (5)	0.0006 (4)	0.0013 (4)	−0.0005 (4)
C3	0.0102 (5)	0.0128 (6)	0.0116 (5)	0.0012 (4)	0.0007 (4)	0.0007 (4)
C4	0.0106 (5)	0.0111 (5)	0.0117 (5)	0.0019 (4)	0.0010 (4)	0.0015 (4)
C5	0.0122 (5)	0.0120 (6)	0.0144 (5)	−0.0019 (4)	0.0036 (4)	−0.0015 (4)
C6	0.0105 (5)	0.0172 (6)	0.0171 (6)	−0.0029 (5)	0.0024 (4)	−0.0028 (5)
C7	0.0101 (5)	0.0177 (6)	0.0172 (6)	−0.0002 (5)	0.0028 (4)	−0.0027 (5)
C8	0.0124 (6)	0.0134 (6)	0.0167 (6)	0.0018 (4)	0.0032 (4)	−0.0030 (5)
C9	0.0105 (5)	0.0120 (6)	0.0144 (5)	−0.0001 (4)	0.0026 (4)	−0.0026 (4)
C10	0.0141 (6)	0.0124 (6)	0.0169 (6)	0.0017 (5)	0.0031 (5)	0.0028 (5)
C11	0.0157 (6)	0.0161 (6)	0.0105 (5)	0.0028 (5)	0.0017 (4)	0.0019 (4)
C12	0.0137 (6)	0.0179 (6)	0.0137 (6)	0.0049 (5)	−0.0004 (4)	0.0014 (5)
C13	0.0246 (7)	0.0209 (7)	0.0232 (7)	0.0059 (6)	0.0034 (5)	0.0080 (5)
C14	0.0133 (6)	0.0154 (6)	0.0137 (5)	−0.0011 (5)	0.0009 (4)	−0.0031 (5)
C15	0.0187 (6)	0.0234 (7)	0.0163 (6)	−0.0020 (5)	−0.0004 (5)	−0.0069 (5)
C16	0.0111 (5)	0.0113 (5)	0.0078 (5)	0.0028 (4)	0.0012 (4)	0.0009 (4)
C17	0.0131 (5)	0.0130 (6)	0.0092 (5)	0.0018 (4)	0.0009 (4)	0.0004 (4)
C18	0.0190 (6)	0.0123 (6)	0.0137 (6)	0.0027 (5)	0.0020 (5)	0.0000 (4)
C19	0.0158 (6)	0.0190 (6)	0.0157 (6)	0.0085 (5)	0.0039 (5)	0.0009 (5)
C20	0.0113 (5)	0.0200 (6)	0.0148 (6)	0.0030 (5)	0.0021 (4)	0.0020 (5)
C21	0.0118 (5)	0.0143 (6)	0.0091 (5)	0.0011 (4)	0.0009 (4)	0.0010 (4)
C22	0.0142 (6)	0.0109 (6)	0.0121 (5)	−0.0001 (4)	0.0016 (4)	−0.0022 (4)
C23	0.0217 (7)	0.0123 (6)	0.0182 (6)	−0.0023 (5)	0.0034 (5)	0.0001 (5)
C24	0.0210 (6)	0.0187 (6)	0.0172 (6)	0.0004 (5)	−0.0009 (5)	−0.0060 (5)
C25	0.0109 (5)	0.0158 (6)	0.0137 (5)	−0.0011 (4)	0.0023 (4)	0.0004 (5)
C26	0.0147 (6)	0.0260 (7)	0.0223 (7)	−0.0025 (5)	−0.0024 (5)	−0.0035 (5)
C27	0.0192 (6)	0.0220 (7)	0.0219 (7)	−0.0027 (5)	0.0065 (5)	0.0046 (5)

### Geometric parameters (Å, º)

**Table:**

N1—C16	1.4412 (15)	C13—H13A	0.9800
N1—C1	1.4794 (15)	C13—H13B	0.9800
N1—C4	1.4928 (15)	C13—H13C	0.9800
N2—C14	1.4662 (16)	C14—C15	1.5274 (17)
N2—C1	1.4675 (14)	C14—H14A	0.9900
N2—C12	1.4745 (15)	C14—H14B	0.9900
C1—C2	1.5741 (16)	C15—H15A	0.9800
C1—H1A	1.0000	C15—H15B	0.9800
C2—C3	1.5449 (16)	C15—H15C	0.9800
C2—C9	1.5445 (16)	C16—C21	1.4162 (16)
C2—C5	1.5456 (16)	C16—C17	1.4215 (17)
C3—C4	1.5402 (16)	C17—C18	1.3974 (17)
C3—H3A	0.9900	C17—C22	1.5260 (17)
C3—H3B	0.9900	C18—C19	1.3853 (18)
C4—C11	1.5324 (17)	C18—H18A	0.9500
C4—C10	1.5406 (17)	C19—C20	1.3800 (19)
C5—C6	1.5340 (17)	C19—H19A	0.9500
C5—H5A	0.9900	C20—C21	1.3978 (17)
C5—H5B	0.9900	C20—H20A	0.9500
C6—C7	1.5260 (17)	C21—C25	1.5222 (17)
C6—H6A	0.9900	C22—C24	1.5361 (17)
C6—H6B	0.9900	C22—C23	1.5390 (17)
C7—C8	1.5271 (18)	C22—H22A	1.0000
C7—H7A	0.9900	C23—H23A	0.9800
C7—H7B	0.9900	C23—H23B	0.9800
C8—C9	1.5307 (16)	C23—H23C	0.9800
C8—H8A	0.9900	C24—H24A	0.9800
C8—H8B	0.9900	C24—H24B	0.9800
C9—H9A	0.9900	C24—H24C	0.9800
C9—H9B	0.9900	C25—C26	1.5337 (17)
C10—H10A	0.9800	C25—C27	1.5370 (18)
C10—H10B	0.9800	C25—H25A	1.0000
C10—H10C	0.9800	C26—H26A	0.9800
C11—H11A	0.9800	C26—H26B	0.9800
C11—H11B	0.9800	C26—H26C	0.9800
C11—H11C	0.9800	C27—H27A	0.9800
C12—C13	1.5186 (19)	C27—H27B	0.9800
C12—H12A	0.9900	C27—H27C	0.9800
C12—H12B	0.9900		
			
C16—N1—C1	117.52 (9)	N2—C12—H12B	109.1
C16—N1—C4	121.44 (9)	C13—C12—H12B	109.1
C1—N1—C4	112.25 (9)	H12A—C12—H12B	107.9
C14—N2—C1	117.99 (9)	C12—C13—H13A	109.5
C14—N2—C12	112.05 (9)	C12—C13—H13B	109.5
C1—N2—C12	113.47 (9)	H13A—C13—H13B	109.5
N2—C1—N1	116.25 (9)	C12—C13—H13C	109.5
N2—C1—C2	115.24 (9)	H13A—C13—H13C	109.5
N1—C1—C2	106.30 (9)	H13B—C13—H13C	109.5
N2—C1—H1A	106.1	N2—C14—C15	111.30 (10)
N1—C1—H1A	106.1	N2—C14—H14A	109.4
C2—C1—H1A	106.1	C15—C14—H14A	109.4
C3—C2—C9	112.07 (10)	N2—C14—H14B	109.4
C3—C2—C5	111.85 (9)	C15—C14—H14B	109.4
C9—C2—C5	107.34 (9)	H14A—C14—H14B	108.0
C3—C2—C1	103.21 (9)	C14—C15—H15A	109.5
C9—C2—C1	114.88 (9)	C14—C15—H15B	109.5
C5—C2—C1	107.46 (9)	H15A—C15—H15B	109.5
C4—C3—C2	107.61 (9)	C14—C15—H15C	109.5
C4—C3—H3A	110.2	H15A—C15—H15C	109.5
C2—C3—H3A	110.2	H15B—C15—H15C	109.5
C4—C3—H3B	110.2	C21—C16—C17	118.98 (11)
C2—C3—H3B	110.2	C21—C16—N1	118.78 (10)
H3A—C3—H3B	108.5	C17—C16—N1	122.22 (10)
N1—C4—C11	112.99 (10)	C18—C17—C16	119.12 (11)
N1—C4—C3	103.28 (9)	C18—C17—C22	117.14 (11)
C11—C4—C3	110.91 (10)	C16—C17—C22	123.73 (10)
N1—C4—C10	110.96 (9)	C19—C18—C17	121.59 (12)
C11—C4—C10	107.56 (10)	C19—C18—H18A	119.2
C3—C4—C10	111.18 (10)	C17—C18—H18A	119.2
C6—C5—C2	113.58 (10)	C20—C19—C18	119.31 (12)
C6—C5—H5A	108.8	C20—C19—H19A	120.3
C2—C5—H5A	108.8	C18—C19—H19A	120.3
C6—C5—H5B	108.8	C19—C20—C21	121.47 (12)
C2—C5—H5B	108.8	C19—C20—H20A	119.3
H5A—C5—H5B	107.7	C21—C20—H20A	119.3
C7—C6—C5	110.69 (10)	C20—C21—C16	119.47 (11)
C7—C6—H6A	109.5	C20—C21—C25	118.29 (11)
C5—C6—H6A	109.5	C16—C21—C25	122.23 (11)
C7—C6—H6B	109.5	C17—C22—C24	112.06 (10)
C5—C6—H6B	109.5	C17—C22—C23	111.79 (10)
H6A—C6—H6B	108.1	C24—C22—C23	108.81 (10)
C6—C7—C8	110.10 (10)	C17—C22—H22A	108.0
C6—C7—H7A	109.6	C24—C22—H22A	108.0
C8—C7—H7A	109.6	C23—C22—H22A	108.0
C6—C7—H7B	109.6	C22—C23—H23A	109.5
C8—C7—H7B	109.6	C22—C23—H23B	109.5
H7A—C7—H7B	108.2	H23A—C23—H23B	109.5
C7—C8—C9	111.79 (10)	C22—C23—H23C	109.5
C7—C8—H8A	109.3	H23A—C23—H23C	109.5
C9—C8—H8A	109.3	H23B—C23—H23C	109.5
C7—C8—H8B	109.3	C22—C24—H24A	109.5
C9—C8—H8B	109.3	C22—C24—H24B	109.5
H8A—C8—H8B	107.9	H24A—C24—H24B	109.5
C8—C9—C2	113.24 (10)	C22—C24—H24C	109.5
C8—C9—H9A	108.9	H24A—C24—H24C	109.5
C2—C9—H9A	108.9	H24B—C24—H24C	109.5
C8—C9—H9B	108.9	C21—C25—C26	111.94 (10)
C2—C9—H9B	108.9	C21—C25—C27	111.11 (10)
H9A—C9—H9B	107.7	C26—C25—C27	109.66 (11)
C4—C10—H10A	109.5	C21—C25—H25A	108.0
C4—C10—H10B	109.5	C26—C25—H25A	108.0
H10A—C10—H10B	109.5	C27—C25—H25A	108.0
C4—C10—H10C	109.5	C25—C26—H26A	109.5
H10A—C10—H10C	109.5	C25—C26—H26B	109.5
H10B—C10—H10C	109.5	H26A—C26—H26B	109.5
C4—C11—H11A	109.5	C25—C26—H26C	109.5
C4—C11—H11B	109.5	H26A—C26—H26C	109.5
H11A—C11—H11B	109.5	H26B—C26—H26C	109.5
C4—C11—H11C	109.5	C25—C27—H27A	109.5
H11A—C11—H11C	109.5	C25—C27—H27B	109.5
H11B—C11—H11C	109.5	H27A—C27—H27B	109.5
N2—C12—C13	112.38 (10)	C25—C27—H27C	109.5
N2—C12—H12A	109.1	H27A—C27—H27C	109.5
C13—C12—H12A	109.1	H27B—C27—H27C	109.5
			
C14—N2—C1—N1	−44.13 (14)	C3—C2—C9—C8	−69.22 (13)
C12—N2—C1—N1	89.84 (12)	C5—C2—C9—C8	53.96 (13)
C14—N2—C1—C2	81.21 (13)	C1—C2—C9—C8	173.39 (10)
C12—N2—C1—C2	−144.82 (10)	C14—N2—C12—C13	−132.20 (11)
C16—N1—C1—N2	−82.53 (12)	C1—N2—C12—C13	91.09 (13)
C4—N1—C1—N2	129.41 (10)	C1—N2—C14—C15	−145.42 (11)
C16—N1—C1—C2	147.71 (10)	C12—N2—C14—C15	79.99 (13)
C4—N1—C1—C2	−0.35 (12)	C1—N1—C16—C21	118.88 (11)
N2—C1—C2—C3	−146.18 (10)	C4—N1—C16—C21	−96.14 (13)
N1—C1—C2—C3	−15.85 (11)	C1—N1—C16—C17	−59.47 (14)
N2—C1—C2—C9	−23.88 (14)	C4—N1—C16—C17	85.51 (14)
N1—C1—C2—C9	106.45 (11)	C21—C16—C17—C18	−2.55 (16)
N2—C1—C2—C5	95.48 (11)	N1—C16—C17—C18	175.80 (10)
N1—C1—C2—C5	−134.18 (10)	C21—C16—C17—C22	176.45 (10)
C9—C2—C3—C4	−97.89 (11)	N1—C16—C17—C22	−5.21 (17)
C5—C2—C3—C4	141.50 (10)	C16—C17—C18—C19	1.60 (18)
C1—C2—C3—C4	26.27 (12)	C22—C17—C18—C19	−177.46 (11)
C16—N1—C4—C11	−10.30 (15)	C17—C18—C19—C20	0.55 (19)
C1—N1—C4—C11	136.35 (10)	C18—C19—C20—C21	−1.75 (19)
C16—N1—C4—C3	−130.19 (11)	C19—C20—C21—C16	0.76 (18)
C1—N1—C4—C3	16.45 (12)	C19—C20—C21—C25	−179.70 (11)
C16—N1—C4—C10	110.60 (12)	C17—C16—C21—C20	1.40 (16)
C1—N1—C4—C10	−102.76 (11)	N1—C16—C21—C20	−177.00 (10)
C2—C3—C4—N1	−26.48 (12)	C17—C16—C21—C25	−178.12 (10)
C2—C3—C4—C11	−147.79 (10)	N1—C16—C21—C25	3.48 (16)
C2—C3—C4—C10	92.57 (11)	C18—C17—C22—C24	54.13 (14)
C3—C2—C5—C6	68.34 (13)	C16—C17—C22—C24	−124.88 (12)
C9—C2—C5—C6	−54.98 (13)	C18—C17—C22—C23	−68.32 (14)
C1—C2—C5—C6	−179.06 (9)	C16—C17—C22—C23	112.66 (13)
C2—C5—C6—C7	57.69 (13)	C20—C21—C25—C26	52.45 (15)
C5—C6—C7—C8	−55.75 (13)	C16—C21—C25—C26	−128.02 (12)
C6—C7—C8—C9	55.59 (13)	C20—C21—C25—C27	−70.53 (14)
C7—C8—C9—C2	−56.42 (13)	C16—C21—C25—C27	109.01 (13)
